# Emerging Trends and Future Opportunities for Battery Recycling

**DOI:** 10.1021/acsenergylett.4c02198

**Published:** 2024-12-13

**Authors:** Jarom G. Sederholm, Lin Li, Zheng Liu, Kai-Wei Lan, En Ju Cho, Yashraj Gurumukhi, Mohammed Jubair Dipto, Alexander Ahmari, Jin Yu, Megan Haynes, Nenad Miljkovic, Nicola H. Perry, Pingfeng Wang, Paul V. Braun, Marta C. Hatzell

**Affiliations:** † Department of Chemical and Biomolecular Engineering, University of Illinois Urbana−Champaign, Urbana, Illinois 61801, United States; ‡ Materials Research Laboratory, University of Illinois Urbana−Champaign, Urbana, Illinois 61801, United States; § Beckman Institute for Advanced Science and Technology, University of Illinois Urbana−Champaign, Urbana, Illinois 61801, United States; ∥ Grainger College of Engineering, University of Illinois Urbana−Champaign, Urbana, Illinois 61801, United States; ⊥ George W. Woodruff School of Mechanical Engineering, Georgia Institute of Technology, Atlanta, Georgia 30332, United States; # Department of Industrial and Enterprise Systems Engineering, University of Illinois Urbana−Champaign, Urbana, Illinois 61801, United States; 7 Department of Materials Science and Engineering, University of Illinois Urbana−Champaign, Urbana, Illinois 61801, United States; 8 Department of Mechanical Science and Engineering, University of Illinois Urbana−Champaign, Urbana, Illinois 61801, United States; 9 School of Chemical and Biomolecular Engineering, Georgia Institute of Technology, Atlanta, Georgia 30332, United States; 10 Department of Electrical and Computer Engineering, University of Illinois Urbana−Champaign, Urbana, Illinois 61801, United States; 11 Institute for Sustainability, Energy and Environment (iSEE), University of Illinois Urbana−Champaign, Urbana, Illinois 61801, United States; 12 International Institute for Carbon Neutral Energy Research (WPI-I2CNER), Kyushu University, 744 Moto-oka, Nishi-ku, Fukuoka 819-0395, Japan

## Abstract

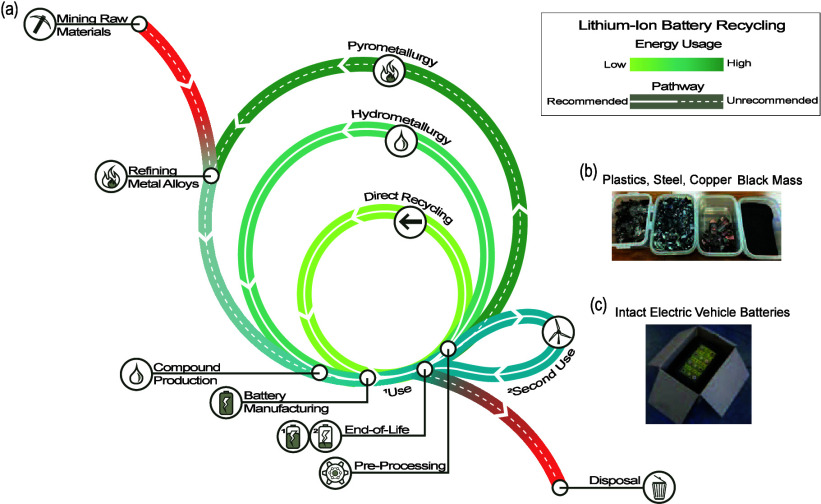

The global lithium-ion battery recycling capacity needs to increase by a factor of 50 in the next decade to meet the projected adoption of electric vehicles. During this expansion of recycling capacity, it is unclear which technologies are most appropriate to reduce costs and environmental impacts. Here, we describe the current and future recycling capacity situation and summarize methods for quantifying costs and environmental impacts of battery recycling methods with a focus on cathode active materials. Second use, electrification of pyrometallurgy and hydrometallurgy, direct recycling, and electrochemical recycling methods are discussed as leading-edge methods for overcoming state of the art battery recycling challenges. The paper ends with a discussion of future issues and considerations regarding solid-state batteries and co-optimization of battery design for recycling.

The lithium-ion battery (LIB) manufacturing industry has experienced tremendous growth in the past decade and is expected to continue to grow over the next decade. The growth of the industry has led to battery manufacturing optimization which has translated into a 82% decrease in the price of LIBs over the past decade.^1^ This price decrease has enabled the use of LIBs in phones, drones, vehicles, appliances, home and grid-scale energy storage, and many other applications. Consequently, the waste industry will soon be inundated with used LIBs. It is estimated that 318 GWh of LIBs will reach their end of life (EOL) by 2030. Of this, approximately half (156.7) GWh is associated with electric vehicle batteries (EVBs).^2^ If the world is not prepared, the push toward decarbonization and the generation of a sustainable economy could result in an unsustainable LIB waste stream.

The LIB recycling industry is still in its infancy, with only 10% of used LIBs recycled. The remaining 90% is disposed of in traditional waste streams.^3^ The recycling rate of mobile phone LIBs, in particular, was less than 5% in 2017. These LIBs have a replacement rate of 12–18 months regardless of actual condition.^3^ Discarded LIBs are not meant to be disposed of in traditional waste streams and are believed to account for 50% of all fires that occur in the waste and recycling industry. These fires pose a risk to employees and cost the North American industry an estimated $2.5 billion annually.^4^ Clearly, improvements in LIB disposal and recycling are needed.

Simultaneously, the extraction of raw materials for LIB manufacturing has significant environmental and social impacts. For example, although electric vehicles (EVs) have a lower carbon footprint than traditional internal combustion engines during their lifetime, the production of EVs can produce up to 68% more emissions than traditional combustion engines, most of which can be attributed to the use of virgin ore-based materials.^5^ There are also significant social impacts, especially with respect to cobalt mining. Today, 60–70% of cobalt is mined in the Democratic Republic of the Congo.^6^ However, there have been links to armed conflict, human rights abuses, illegal mining, and harmful environmental practices regarding cobalt extraction in Congo.^7^

Much of the mining and manufacturing of LIBs is located in varying regions, incurring transportation costs and challenges, and inducing supply chain risks.^8,9^ Approximately 60% of the global lithium reserves are located in Chile and Australia.^10^ Most LIBs are manufactured in China and 93% of the global demand for LIBs stems from China, the United States, and Europe.^11^ Maintaining a LIB life cycle within a single country could reduce the transport costs of recycling by up to 70%.^12^ Many regions are attempting to address these issues by implementing plans to generate a circular economy for LIBs within that region. An example of this is the “The National Blueprint for Lithium Batteries 2021–2030” which was developed in the United States. This plan encourages the recycling of LIBs especially for the recovery of cobalt and nickel.^13^

Here, we aim to provide an overview of emerging trends and future opportunities for battery recycling. We describe the current recycling capacity and future changes to be implemented for creating a cyclic LIB economy. We identify methods for identifying which recycling processes decrease cost and environmental impact of battery recycling. We discuss novel recycling methods that aim to improve sustainability, cost, and throughput of LIB recycling with a focus on the cathode active materials. We end with a discussion of future considerations regarding battery recycling as battery production potential expands in different directions including solid-state batteries and co-designing new battery architectures to support sustainability.

## Emerging Trends in Battery Recycling

**Figure 1:**
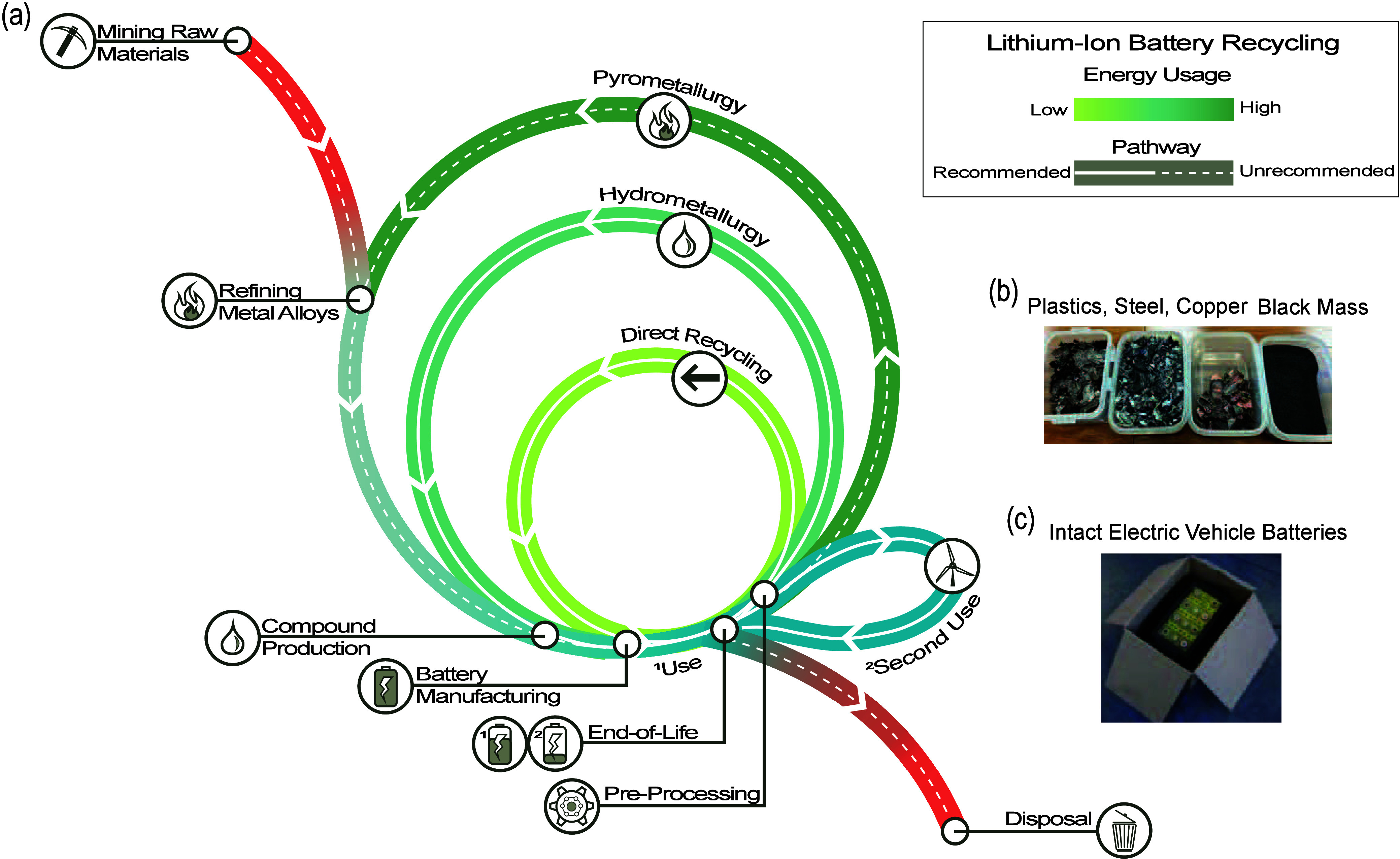
(a) Schematic of a LIB circular economy. (b) Photo of separated materials following mechanical preprocessing from an announcement for the Recyclus group.^22^ (c) Photo of an intact LIB from an eBook being shipped which contains comprehensive packing and shipping strategies.^23^ Inspired by the ReCell Center diagram from an article concerning the recycling of critical materials in the LIB supply chain.^24^

Raw materials for battery cathodes, such as cobalt and nickel, are essential for the global energy landscape. This is reflected in battery material costs where the cathode constitutes the most expensive part of battery cells.^14−19^ The traditional life cycle of a LIB is shown in Figure 1.^20^ The cycle begins with the extraction of raw materials that are processed through metal refining and compound production and then through multiple steps converted into secondary batteries for use by the consumer. In recycling facilities, LIBs are sorted, disassembled, and preprocessed prior to materials recovery. Preprocessing separates the EOL LIB into the predominant bulk materials that include copper, plastics, steel, and black mass (Figure 1b). The black mass contains the active materials of the battery. The black mass is then processed for material recovery through pyrometallurgical or hydrometallurgical processes with direct recycling processes being introduced in the near future. As the LIB lifecycle moves toward a circular economy, a second use can be added to reallocate the used EOL LIBs (Figure 1c) to a lower capacity application before being transported to a recycling facility.^21^

**Figure 2:**
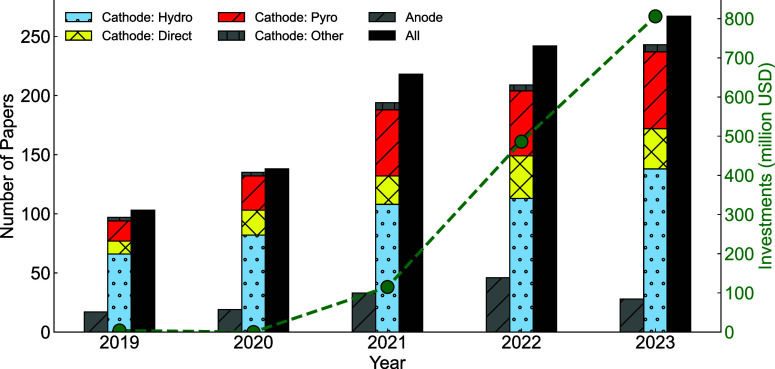
Peer-reviewed and conference works from Web of Science on LIB recycling methods from 2019 to 2023 organized on the bar chart by the materials being recovered and the method through which cathode materials are recovered. The amount of global growth-stage venture capital investment in millions of USD^28^ is shown by the line graph.

Unless economically viable recycling practices are adopted, increased battery production will continue to result in considerable waste.^25^ There is a growing market for battery recycling, with estimates suggesting that the market value could exceed 20 billion US dollars by 2030.^26,27^ The growing market and interest in cathode material recycling are reflected in the increasing number of battery recycling articles published and the increase in global growth-stage venture capital investments^28^ over the past five years (Figure 2). We note, we do not highlight works that focused on the delamination of electrode materials, the recycling of non-lithium-based batteries, the upcycling/recycling of battery materials into non-battery materials, or review articles of any kind in the results of Figure 2. The recovery of cathode active materials largely dominates the battery recycling academic literature. Anode recycling papers account for less than 20% of academic papers over the past 5 years and even show a slight decrease in publications in 2023. Anode materials also account for a much smaller portion of battery costs than cathode materials.^14−19^ For these reasons, we will focus mainly on the cathode active material in this work. We recommend other reviews to find discussions on anode materials.^29,30^

The most common industrial processes for recovering cathode materials, pyrometallurgy and hydrometallurgy, require several steps with various acids, bases, and/or redox controlling agents to remove, separate, and recover each element.^25,31−40^ The cost effectiveness of recovering vital materials from battery cathodes is a major bottleneck to battery recycling implementation.^41,42^ The challenges associated with battery recycling are only magnified when considering the environmental and health impacts of the recycling process. Byproducts from the recycling processes range from greenhouse gas emissions like carbon dioxide to toxic gas creation like chlorine gas and SO_*x*_.^17,43−46^ To be widely adopted, current battery recycling methods must decrease in cost and reduce their harmful emissions to the point of being more advantageous compared to mining new raw materials.

All current battery recycling methods have pitfalls. There are three areas of improvement that are foremost to consider as efforts progress to improve the battery recycling industry: recycling capacity, cost, and environmental impact. Recycling capacity impacts the recycling industry as a whole. Battery recycling capacity includes factors such as transportation, sorting, disassembly, and preprocessing of EOL batteries. Only after these factors are addressed can one consider battery recycling processes. The cost of battery recycling is highly dependent on which battery recycling method and process is used. The same is true of the emissions generated and harmful byproducts produced. As such, all new methods should be evaluated through the lens of cost effectiveness and impact on humans and the environment.

### Recycling Capacity

The current global capacity for battery recycling is approximately 200 kt/year^11^ and concentrated in Asia (nearly 60% of the total capacity) with Europe and North America accounting for roughly 36% and 6% respectively. It is predicted that the global capacity will increase to nearly 1200 kt/year causing a reduction in the primary supply requirement for critical battery materials by 12% by 2040.^47^ One method for increasing overall battery recycling capacity is to change from few large facilities to many small ones. For example, commercial recycling facilities tend to focus on large, centralized materials recovery, as these tend to be batch processes that have significantly lower operational and material costs as they increase in scale. Unlike materials recovery, mechanical preprocessing does not necessarily follow economies-of-scale and can be applied to smaller facilities in urban environments.^48^ Movement to urban environments could also decrease the emissions caused by battery collection which accounts for 70% of total battery recycling transportation CO_2_ emissions.^43^ This is advantageous because as the capacity to recycle LIBs improves, EOL LIB collection must also increase.

Improved infrastructure for collecting and recycling battery materials is vital to the “Net Zero Emissions by 2050 Scenario” released by the International Energy Agency. According to the Scenario, LIB recycling collection rates should increase from 45% in the early 2020s to 80% by 2040.^49^ To reach these goals, changes to battery recycling infrastructure are vital. Current battery transportation is difficult as EOL LIBs are often considered hazardous waste. Designating an intact LIB as a hazardous material adds both complexity and additional costs to the logistics of transportation of the EOL LIB.^25,50,51^ However, after the black mass is separated through preprocessing, only the black mass is classified as a hazardous material, and the copper, plastic, and steel recovered during this process can be transported to their traditional recycling industries without additional regulatory policies and transportation costs.^17^ Black mass can make up approximately 50% of the total mass of an intact LIB.^52^ Therefore, performing the preprocessing step in small distributed facilities closer to the end use of LIBs would reduce the amount of hazardous material in transit and consequently the transportation costs associated with LIB recycling, further incentivizing decentralization.^53,54^ Recognizing this issue, machine learning has been utilized to show how LIB recycling transportation can be optimized in California by using different sizes of battery recycling plants in different locations as a function of the amount of predicted EV battery waste in the area.^20^ Other work found decentralized dismantling and preprocessing facilities in Europe were found to reduce costs by half and were more economical even when accounting for amortization costs of the new facilities.^54^

### Cost

**Figure 3:**
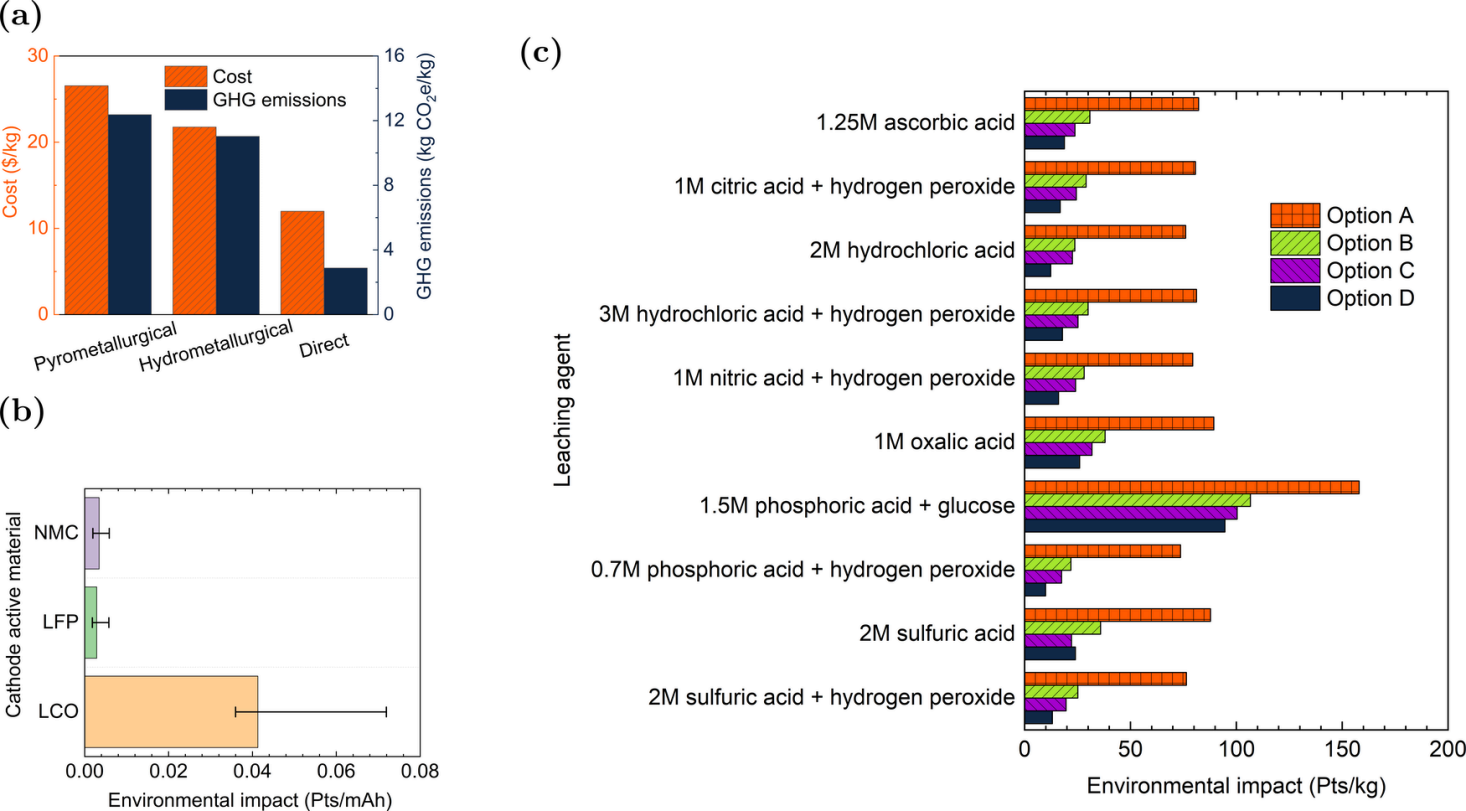
Use of cost and environmental impact assessments in battery recycling. (a) Comparison of hydrometallurgical, pyrometallurgical, and direct recycling processes for NMC111 cathodes in terms of cost and GHG emissions. (b) Comparison of different cathodes through hydrometallurgical recycling. The black bar represents the environmental impact as a function of the lifespan of the cathode active material. (c) Comparison of different hydrometallurgical recycling processes for LCO cathodes. Different options represent different process flow diagrams.

There are many costs incorporated with battery recycling, including transportation costs. However, the costs of battery recycling methods are most commonly compared using the operating cost. The operating cost of each process is split into several criteria, with the two largest costs generally coming from the cost of input materials and the cost of energy. Lab-scale analyses have been performed for battery recycling processes^31^ and have shown how new methods are comparable in terms of cost per kg waste cathode. However, tools which enable industrial-scale comparison do exist. EverBatt,^55^ an Excel-based tool created by the Argonne National Laboratory, is commonly used to compare the costs of current and new battery recycling processes^56^ on an industrial scale in terms of both operating costs and capital costs. This tool has been used to show how recycling through hydrometallurgy and pyrometallurgy are 33–53% less costly than mining new materials for several battery cathode materials.^57^ Cost can also be calculated for a specific process and material. Figure 3a shows the costs of different recycling processes for NMC111 cathodes, which shows the advantage of direct recycling compared to hydrometallurgical and pyrometallurgical processes.^58^

### Environmental Impact

Of increasing importance is the environmental impact of battery recycling processes. The use of environmental impact, or life cycle assessment (LCA), has grown in popularity as the impact of industrial battery recycling has increased. However, the method for performing a LCA varies. EverBatt is commonly used to determine the environmental impact of a certain process by calculating the quantity of greenhouse gases, volatile organic compounds, particulate matter, and other similar emissions. The environmental impact analyzed through EverBatt is shown as greenhouse gas (GHG) emissions. The GHG emissions of different recycling processes for NMC111 cathodes are compared^58^ in Figure 3a. This shows direct recycling has a lower environmental impact compared to other methods. The ReCiPe model^59^ is another tool which has been used for LCA. The ReCiPe model, like the EverBatt tool, determines the amount of compound emissions, but then sorts the emissions into separate damage pathways that end in three main criteria: damage to human health, damage to ecosystems, and damage to resource availability. The ReCiPe model can be used for different comparisons, e.g., a comprehensive comparison of the recycling of different cathode active materials considering their specific capacity and battery lifespan,^60^ as shown in Figure 3b. This provides needed insights for material development. Also, the ReCiPe model can be used for the comparison of different hydrometallurgical recycling processes and leaching agent choices,^60^ as shown in Figure 3c, allowing for process to process comparison within a recycling method.

Binders, particularly the popular poly(vinylidene fluoride), PVDF, used in battery cathode production constitute a sustainability hurdle.^50,61^ The binder, while necessary to hold the cathode active material together, complicates the batteries’ fast disassembly and can produce environmentally harmful species during recycling. Binder is traditionally dealt with using three methods: solvent dissolution,^62^ mechanical processing,^63^ and thermal decomposition. Thermal processing of PVDF results in the formation of toxic compounds, including HCN, HF, CH_4_, HCHO, COF_2_, SiF_4_, HNCO, CO, CO_2_, and nitrogen oxide, causing pollution with fluorine-containing compounds being particularly problematic.^64,65^ HF also reacts with cathode materials, causing delithiation after recycling,^45^ and lower temperatures used during the hydrometallurgical recycling processes could favor the incomplete decomposition of perfluoroalkyl substances (PFAS) and/or the formation of new fluorinated compounds.^66^ Recognizing these hazards and performance issues, PVDF has been replaced with binders that are easier to recycle in anode materials and in LFP cathodes. LFP accounts for 40% of EV battery production in the world as of 2023 with the largest congregation in China where 67% of electric vehicles use the LFP battery chemistry. However, PVDF continues to be the main binder for nickel-containing cathodes. This is because other binder polymers are water-soluble and cause issue when interacting with the moisture-sensitive nickel-containing active materials.^67^ Nickel-containing cathodes account for the remaining 60% of EV battery production worldwide and 93–94% of the electric vehicles in the United States and Europe as of 2023.^28^ As such, more research on how to safely and sustainably account for PVDF in nickel-containing cathodes is required and will continue to be required in the coming years.

Efforts exist to make changes to current battery recycling methods at every step of the process to reduce the cost and the environmental impact of battery recycling methods. For example, past work has analyzed efforts made at the beginning of the battery recycling process by focusing on the delamination of cathode materials from their current collectors. These efforts not only reduce separation costs and maximize cathode material recovery, but also have important environmental implications in relation to the removal of PVDF.^61^ Current recycling methods struggle in terms of both cost and adverse environmental impact and must be improved to meet demand. Future technologies proposed to overcome these challenges are explored here.

## Opportunities for Battery Recycling

### Second Use

An EOL processing technique recently gaining attention is the concept of second use, also known as reuse. Due to planned obsolescence, many consumer electronics that use LIBs or EV batteries enter the waste stream roughly 8–10 years after their original manufacturing date with a state of health of approximately 70–80% of the original battery capacity.^54^ EOL LIBs from many other industries also enter the waste stream with a large majority of their original capacity still intact. Directing these partially spent LIBs to other applications that do not require full original capacity could partially offset the massive influx of EOL LIBs while simultaneously supplying immediate electric storage capacity, decreasing the demand for manufacturing of new LIBs for these applications, and providing LIBs without the need for extensive recycling. The National Renewable Energy Laboratory (NREL) reported reuse of plug-in electric vehicle (PEV) LIBs for grid-connected combustion turbine peaker plants would be economically and environmentally beneficial by saving end of service costs of LIBs and cost of peaker plant operation, lowering fossil fuel use and greenhouse gas emissions.^68^ Also, a study estimated 73–100% decrease in the accumulative new battery demand in 2050 in China if all EOL electrical vehicle batteries are put into second use, which highlights its importance.^69^ Not only that, redirecting EOL LIBs from the LIB recycling industry could give the industry time to increase their recycling capacity and improve existing processes, reducing energy requirements, streamlining process flows, optimizing transportation networks, and implementing novel methodologies that produce fewer toxic byproducts.

Potential second-use applications can be classified by their mobility or energy storage requirements, with the current focus being on stationary applications.^70^ Stationary applications include providing capacity for backup energy storage for residential use or storage for renewable energy such as wind or solar. Semistationary uses apply to applications which do not require long-term storage, such as construction sites or mobile offices. Mobile uses include the reuse of LIBs in a similar application, such as EVBs in golf carts or other small motorized vehicles. Categorization by energy requirement generally corresponds to small, medium, or large-scale storage ranging from residential use to commercial or industrial applications.^70^ Currently, questions remain about what categories of energy requirement should be prioritized when considering second-use applications. Competing research has found that the most efficient use is in large-scale stationary storage, and not smaller, residential-scale storage, and vice versa.^70−72^ Even with the aforementioned advantages, it does remain an open question if the benefit required for second-use will be worth the investment versus recycling EOL batteries into modern high-performance batteries. To perform more accurate comparisons, real application cases are required as most of the current second-use LIB studies are based on assumptions and modeling due to the lack of real life cases.

### Electrification of Pyrometallurgy and Hydrometallurgy

**Figure 4:**
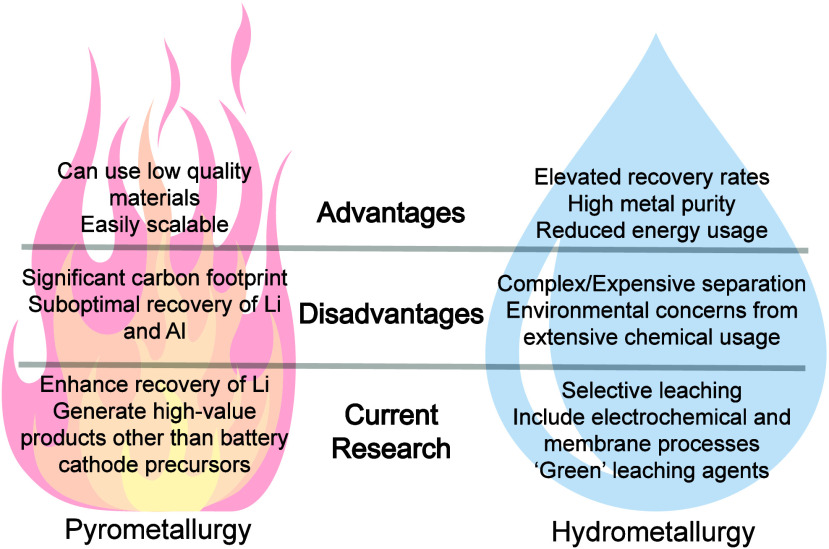
Advantages, disadvantages, and current research surrounding pyrometallurgy and hydrometallurgy recycling processes.

Pyrometallurgical and hydrometallurgical processes are currently the most popular methods for recycling battery materials. The use of these methods is well covered in other reviews^41,73^ and, as such, will not be covered here. However, there are currently many efforts being explored to improve hydrometallurgical and pyrometallurgical processes due to their individual disadvantages (Figure 4).

Of particular interest is the electrification of these processes. For example, flash joule heating (FJH) has been used to improve pyrometallurgical processes. FJH is a fast and energy-efficient heating method for metal extraction that can improve performance and reduce the environmental impact of conventional pyrometallurgical techniques. By applying a pulsed direct current to the black mass with 80 V for 110 ms, extraction of lithium and transition metals from the initial black mass was enhanced by about 1000 times even using diluted acid such as 0.01 M HCl.^57^ The enhancement was hypothesized to be from the organic components of the SEI layer degrading and the oxidation states of the metal elements in the insoluble compounds lowering. This in turn improved the contact of the black mass with the acid solution. Furthermore, FJH offers a way for efficient and low-energy battery direct recycling.^74^ By using a rapid roll-to-roll manufacturing process that passes EOL cathode materials and precursor through a flash heating zone for only eight seconds, regenerated LCO cathode materials were obtained for reuse. The regenerated LCO showed electrochemical performance similar to the original LCO materials. These studies demonstrate the potential of electrothermal processes for the fast and economical recovery of valuable cathode materials. A detailed LCA shows that the FJH method reduced HCl consumption by 87% and water consumption by 26% when compared to hydrometallurgical methods. Furthermore, the LCA showed a 26% reduced energy consumption and 38% reduced greenhouse gas emissions when compared to pyrometallurgical methods.^57^ Similar FJH processes have been used to recycle battery materials, such as flash graphene production.^75,76^ The scalability of the method needs to be analyzed, but electric heating provides a promising route for battery recycling.

Hydrometallurgy has also begun implementing electrified processes to decrease dependency on costly separations. Leveraging the distinctive attributes of ion exchange membranes, electrodialysis (ED) emerges as a promising method for concentrating, separating, and selectively recovering metals from wastewater or metal leachate.^77^ ED technology boasts advantages such as continuous operation, scalability, decarbonization, and user-friendliness, which makes it appealing to the waste LIB recycling sector. This approach reduces the volume of leaching and waste solutions and employs ion-exchange membranes in tandem with techniques such as electroplating to enhance metal selectivity.

Lithium recovery from leachates through ED has been investigated, employing *N*-methyl diethanolamine as a regenerable catholyte for CO_2_ capture.^78^ Following nickel and cobalt separation, the proposed method achieved high purity lithium recovery from LIB leachate, which mainly contained lithium and manganese, with only CO_2_ consumption. The process utilized a monovalent selective cation exchange membrane (CEM) for the separation of lithium and transition metals. In catholyte, an amine-based CO_2_ capture solution facilitated lithium recovery through Li_2_CO_3_ precipitation. However, the Li_2_CO_3_ yield of 48% falls short of industrial requirements, necessitating additional electrodeposition for Mn^2+^ recovery. A study focused on LiCoO_2_ cathode materials using 0.1 M HCl^79^ compared the leaching-electrodialysis process using a three-compartment ED unit with cation exchange membranes. However, the commercial CEM selectivity resulted in recovery of 62% lithium and 33% cobalt in the catholyte. The future application of ED for battery recycling requires further exploration and is highly dependent on the development of membranes and membrane selectivity.

For cost-effective purification, researchers are striving to create selective materials that can be adapted to various battery recycling methods. Researchers have enhanced poly(vinyl chloride) films using ethylenediamine and then introduced 5-chloro-8-hydroxyquinoline (5C8Q) chelating agents through two synthesis routes to improve selectivity.^80^ The modified films were employed as selective CEMs for the separation of cobalt, lithium, and nickel, in comparison with those of commercial CEMs. ED-based separation resulted in recovery of 60% cobalt in 1 h, while nickel and lithium showed recoveries of 18% and 0.2%, respectively. These chelating-agent-mediated materials offer a sustainable approach by enhancing targeted ion transfer, enabling the recovery of individual ions with high-purity products, and facilitating the creation of zero liquid-discharge units. Others developed a novel membrane-based hybrid system for the recycling of EOL LIBs, which applied a nanofiltration membrane (VNF2) to obtain a high rejection rate (>92.5%) of Ni^2+^, Co^2+^, and Mn^2+^, and a high permeating rate of Li^+^ (>89.6%).^81^ The utilization of membrane-based processes for battery recycling is booming, including examples outside of ED such as nanofiltration and vacuum membrane distillation. However, these applications are on the lab scale, but with further development of membrane properties, the future commercial applications are promising.

### Direct Recycling

Direct recycling is a novel LIB recycling method and is currently at the experimental and start-up phase. Unlike pyrometallurgy or hydrometallurgy, direct recycling methods do not separate the individual elements of a battery, allowing the cathode and anode to retain their original chemical composition. Additionally, direct recycling does not have to be performed in large scale or a batch process to be economical and could be applied to smaller facilities in urban environments.^48^

Direct recycling requires rigorous sorting methods to separate LIBs by chemistry and then mechanical pretreatment to separate the casing, current collectors, and electrode materials of the LIBs. Optionally, prior to mechanical pretreatment, the separation of the liquid electrolyte (solvent and lithium salt) can be achieved using supercritical CO_2_ or a thermal treatment process. The remaining cathode material is then relithiated prior reuse in the LIB manufacturing process.^82^ The main benefit of this method is that the energy requirements are 80–90% lower than hydrometallurgical and pyrometallurgical processes.^83^ Furthermore, toxic materials are not used, enabling reduction of pollution, toxic wastes, and cost relative to traditional recycling. Lastly, the direct recycling process is a simple process.

There are still challenges surrounding direct recycling. The direct recycling processes are tailored to specific electrode chemistries and cannot simultaneously process various cathode compositions. Hence, rigorous sorting by battery cathode chemistry (i.e., LCO, LFP, different NMCs, etc.) is required and stands as a significant difficulty in scaling this process to an industrial scale.^3^ As the cathode compositions continue to change, older cathode chemistries may no longer be useful for remanufacturing.^17^ For example, NMC cathodes, which are increasingly being utilized for EVs, undergo more complicated degradation mechanisms when compared to LCO, making direct recycling a more difficult pathway for regeneration.^17^ Additionally, recovered battery materials typically have impurities and degraded surface properties correlated with poor electrochemical performance, decreasing the effectiveness of direct recycling. However, there has been some success in achieving performance equivalent to newly manufactured LIBs by processing materials with a high-temperature thermal treatment.^84^ Because this process has only been performed at the laboratory scale, it is unclear whether these materials will maintain the necessary electrochemical performance over the long-term when compared to traditional recycled or manufactured LIBs.^82,85^

### Electrodissolution and Deposition

Research has moved beyond simply implementing electrified steps into traditional battery recycling methods and has begun utilizing fully electrochemical methods for recycling. Electrochemical methods for recovering battery materials have arisen as a solution to combat two pitfalls of current recycling methods: cost and negative byproduct formation. Battery recycling cost is decreased through electrochemical methods primarily through the removal of costly leachants and redox agents and/or by replacing expensive separation or cathode production steps. For example, electro-dissolution has been utilized to recover lithium in the form of lithium carbonate (Li_2_CO_3_) at a purity above 99 wt % from LFP cathodes.^40^ However, lithium is not the only recoverable element. Research has gone one step further and electro-dissolution has been used to recover both lithium carbonate and cobalt in the form of cobalt metal or cobalt oxide (CoO).^86^ Electrodeposition has also been used to simultaneously recover both cobalt and nickel metal at a purity near 96% and 94%, respectively, from solutions made by chemically leaching NMC cathodes.^31^ Additionally, electrodeposition can be used to directly create new LCO cathodes.^87^ Usage of these methods not only negates the need for costly and potentially hazardous chemicals and processes but is also a novel tool to further electrify chemical systems in an effort to create a more sustainable chemical industry.

There remain aspects of electrochemical recovery that have been untapped. Unlike some conventional battery recovery methods, electrochemical methods have yet to be used to recycle battery waste that contains multiple battery chemistries. The usage of electrochemical methods to recover other non-cathode materials has also not been explored. Although electro-dissolution and electrodeposition are commonplace in the metallurgy industry, the impact of the application of these tools in terms of both cost and life cycle impact in the battery recycling industry has yet to be explored. New research in this area could prove invaluable in creating a more circular and cost-effective energy storage industry.

## Future Considerations

As battery research and the battery industry continue to evolve and grow, battery recycling research and industry must also change and expand. Battery research efforts are pushing for the introduction of new battery chemistries and structures, with examples including the introduction of an all-solid-state battery design. The ever-increasing demand for batteries is also encouraging efforts to alter battery packaging and production to be codesigned with recyclability and life cycle in mind. The following contains considerations for battery recycling to keep up with future battery production and usage.

### Solid-State Batteries

Solid-state batteries (SSBs) are of significant interest for applications including EVs because of potential safety and energy density advantages over conventional LIBs containing liquid electrolytes. These features are achieved by replacing liquid electrolytes (LEs) with solid-state electrolytes (SSEs) and the utilization of lithium metal anodes.

A notable difference in the recycling of SSBs is the presence of SSEs. Currently, organic solvent-based LEs are usually removed during the recycling process.^88^ However, SSEs will be recycled differently depending on their compositions with oxide, sulfide, and halide-based chemistries all under consideration.^89^ Depending on the composition, recycling SSEs presents varied challenges. For example, sulfide-based SSEs are hygroscopic and can generate toxic H_2_S gas in the presence of water.^90^ Furthermore, their mechanical pliability leads to a low separation efficiency.^91^ To address the challenges of sulfide-based SSE, a pretreatment method known as dissolution–precipitation has recently been explored. This process involves the dissolution of sulfide SSEs using polar solvents followed by precipitation of the SSE by evaporating the solvent. This enables separation of the SSE from other cell components which are not soluble in the polar solvents.^92^ There is ongoing work to find the best organic solvents to use along with the best method for then recovering a SSE with high ionic conductivity as some methods create low ionic conductivity materials.^93^ Problems can be found with other compositions such as oxide-based SSEs, where, for example, the popular garnet-type Li_7_La_3_Zr_2_O_12_ (LLZO) may cause recycling complications simply due to the number of different elements involved. When recycling SSEs containing multiple metal elements, selecting suitable leaching and precipitation environments becomes crucial to avoid unfavorable elemental distribution during the coprecipitation, which in turn requires additional steps to recover individual components. In light of these considerations, simpler and greener recycling technologies are gaining more attention. For example, recently a hydrometallurgical process was developed using an organic acid as a leaching agent to recover Li_6.5_La_3_Zr_1.5_Ta_0.5_O_12_ (LLZTO).^94^ In another report, short-circuited LLZTO SSEs containing Li dendrites were ball milled, mixed with fresh LLZTO and sintered, leading to the formation of new LLZTO SSEs without the need for leaching agents.^95^

### Co-design Consideration

The wide variety of battery designs and chemistries cause additional obstacles for recycling.^25,50^ Without standardization of battery design, collected EOL batteries typically require manual presorting before recycling treatments. Current options such as the Optisort system, which uses a computer vision algorithm to identify battery labels and sort batteries by their chemistries,^96^ could be useful for automation of battery disassembly, but even these systems will require pack disassembly and presorting before use.^25^ Current secondary batteries were not developed with recyclability considerations paramount. Without external intervention, it might not be economically attractive for manufacturers to design secondary batteries to improve recyclability, but as the volume of EOL batteries grows this may change. Because SSBs are still in the development phase, there is a great opportunity to co-design cells and stacks for recycling before specific designs are finalized.

The physical design of cells has mainly focused on maximizing the power and energy density with little attention paid to their serviceability and recyclability. This also applies to battery packs made up of these cells, where packaging cost, density, and thermal performance are key priorities. However, for SSBs in the development phase, it may be beneficial to explore packaging designs that support easier mechanical disassembly, simplifying future recycling efforts. Currently, there are three main types of packaging for secondary batteries: cylindrical, prismatic, and pouch cells. However, it is still uncertain whether these packaging formats will offer the optimal balance between recyclability, cost, and performance for SSBs. Additionally, the importance of battery thermal management and design for recyclability provides an opportunity for cell and battery pack design. Perhaps future cell and pack designs could utilize aspects of the battery thermal management system to improve recyclability. Newer batteries could, for example, contain internal cooling channels that could also be used to flow etchants through them to enable an “inside out” recycling process which would eliminate the need to disassemble a pack prior to recycling, saving time and money while also improving safety. However, this may require changes to the selection of materials in cells, such as the separators, since current polyolefin-based separators are highly resistant to degradation and can present complications for etching processes.^97^ A switch to water-based or biobased binders could simplify recycling, as these materials would allow electrode components to be easily separated through water washing.^98,99^

### To Recycle or Not to Recycle

The increasing demand for lithium-ion batteries faces limitations in the supply of battery metals from mining. This imposes significant pressure on environmental, economic, national security, and ethical considerations as the current LIB supply chain originates from the extraction and processing of raw minerals. The current distribution of essential battery minerals also presents potential geopolitical challenges. Even at cost parity with mining raw materials, battery recycling emerges as an attractive approach for obtaining the minerals required for battery manufacturing.

**Figure 5:**
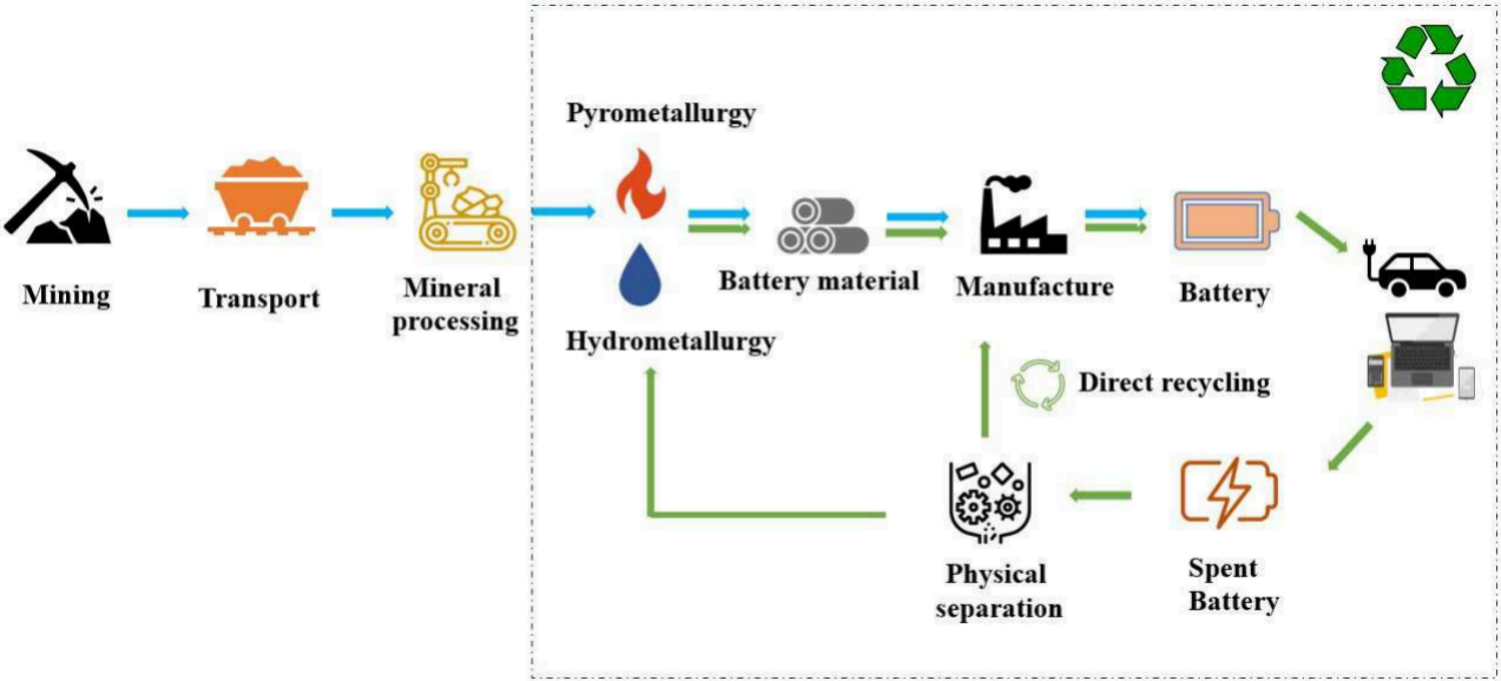
The supplementary role of battery recycling in battery manufacturing from mined resources.

The attractive nature of EOL battery recycling holds across multiple dimensions, including energy consumption, greenhouse gas emissions, and water usage.^100^ A comparison was conducted between conventional mining and recycling of LIB supply chains, utilizing hydrometallurgical and pyrometallurgical processes of a recycling company (Redwood Materials). This is depicted in Figure 5. Their findings revealed that the circular supply chain exhibited significantly lower environmental impacts, ranging from 86% to 99%, compared to conventional methods, with a remarkable 335% reduction in greenhouse gas emissions. Notably, refinement (involving material extraction and transport) accounted for less than 5% of the environmental impacts in circular LIB supply chains, contrasting with 31% in the conventional counterpart.

However, there are still situations where recycling may not be the best option. For example, LFP, a battery chemistry growing in popularity for EVs, is economically a challenge for battery recycling as it does not contain high-value metals like nickel or cobalt. This makes recycling this battery chemistry unprofitable through conventional recycling methods.^11^ Very similar trends exist for lithium manganese oxide where the net loss from recycling would be between $10-$20 per kWh recovered.^12^ Emissions from battery recycling can also exceed those of mining pristine materials. LFP battery recycling methods currently release more emissions than mining with some recycling methods producing up to 2 kg more of CO_2_ per kg of battery than mining. If the wrong recycling approach is selected, recycling battery materials that contain high value products like NMC and lithium nickel cobalt aluminum oxide may still produce more emissions than mining with emissions being highly dependent on the recycling method and battery packaging.^43^

As battery recycling advances, industry and academia must identify and focus on issues preventing recycling from being preferred over mining new materials. For example, using standard EverBatt 2020 models of hydrometallurgical, pyrometallurgical, and direct recycling processes in the United States on LCO battery packs, we find that greenhouse gas emissions of each recycling step vary considerably with the battery recycling procedure. Pyrometallurgy emissions come mostly from the actual process (61%) whereas hydrometallurgy emissions come almost purely from the production of materials going into the hydrometallurgy process (74%). Direct recycling emissions differ from both as most emissions come from energy put into the process (64%). Looking deeper we find that the process emissions from pyrometallurgy primarily come from the combustion of the materials used in the pyrometallurgy process (88% of process emissions), motivating research to either improve the combustion process to minimize emissions or alter the process completely to no longer require combustion. Similar analyses can be done on hydrometallurgy showing the majority of the emissions come from the use of sodium hydroxide and hydrogen peroxide (57% and 35% of emissions respectively) inspiring the usage of different chemicals or alterations to the leaching process to minimize the need for chemical agents. Direct recycling emissions are dependent on the source of energy used (i.e., electricity, diesel, natural gas) for upstream electricity production and on-site fuel combustion for the process; this motivates research toward lower energy requirements and changes to optimize the process for low carbon energy sources. These analyses can be extended to other aspects of battery recycling including transportation. Transportation produces between one-third and one-fourth of the emissions of the recycling process with most emissions coming from transport from battery disassembly sites to the recycler (44%) and from the recycler to the cathode producer (24%). A similar analysis could, and should, be done to identify other major recycling costs. These analyses will be dependent on battery design and can inspire recyclablility-oriented battery architectures. The analyses can also inspire improvements to current recycling processes or the development of entirely new recycling processes. Should recycling methods fail to be environmentally sustainable and cost-efficient, methods to effectively convert EOL battery materials into materials for other applications such as electrocatalysis, water electrolysis, supercapacitors, and Zn-air batteries^101−105^ could be explored. For example, an upcycled LCO cathode could be utilized not only for hydrogen evolution reactions (HER) and oxygen reduction reactions (ORR) but also in other electrochemical systems, such as carbon dioxide reduction and nitrogen fixation, thereby expanding its applicability in sustainable energy technologies.^101^

As long as batteries continue to be used, battery recycling will be an important topic. Cost and environmental impact analyses tools are valuable to ascertain the benefits of new battery designs and recycling methods. These tools also assist in determining necessary changes to infrastructure and battery consumer behavior to fully realize the benefits of large-scale battery recycling. However, all this work must be supported by research that improves overall battery recycling capacity. Further changes to battery recycling methods will be required to create a sustainable and cost-effective battery economy as the battery industry continues to grow and evolve. While the battery and recycling technologies presented here are not exhaustive, we hope they serve to show areas of opportunity for academic and industrial research on battery recycling.
